# Clinical outcome of prophylactic oophorectomy in BRCA1/BRCA2 mutation carriers and events during follow-up

**DOI:** 10.1038/sj.bjc.6601692

**Published:** 2004-03-16

**Authors:** R I Olivier, M van Beurden, M A C Lubsen, M A Rookus, T M Mooij, M J van de Vijver, L J van't Veer

**Affiliations:** 1 Department of Gynaecology, The Netherlands Cancer Institute/Antoni van Leeuwenhoek Hospital, Plesmanlaan 121, 1066 CX Amsterdam, The Netherlands; 2 Department of Gynaecology, Academic Medical Centre, Meibergdreef 9, 1105 AZ Amsterdam, The Netherlands; 3 Department of Epidemiology, The Netherlands Cancer Institute/Antoni van Leeuwenhoek Hospital, Plesmanlaan 121, 1066 CX Amsterdam, The Netherlands; 4 Department of Pathology, The Netherlands Cancer Institute/Antoni van Leeuwenhoek Hospital, Plesmanlaan 121, 1066 CX Amsterdam, The Netherlands; 5 Family Cancer Clinic, The Netherlands Cancer Institute/Antoni van Leeuwenhoek Hospital, Plesmanlaan 121, 1066 CX Amsterdam, The Netherlands

**Keywords:** fallopian tube carcinoma, PPSC, BRCA1 mutation, BRCA2 mutation, prophylactic salpingo-oophorectomy

## Abstract

A retrospective study was performed to assess the histopathologic findings in high-risk women undergoing bilateral prophylactic (salpingo)-oophorectomy. The medical files of BRCA1 or BRCA2 mutation carriers and members of a hereditary breast/ovarian cancer (HBOC) family, who had undergone prophylactic surgery, were reviewed. In all, 38 women underwent a bilateral oophorectomy (26 BRCA1, three BRCA2 and nine HBOC, respectively). A total of 90 women underwent bilateral salpingo-oophorectomy (58 BRCA1, six BRCA2, one BRCA1 and 2, 25 HBOC, respectively). At the time of salpingo-oophorectomy, five of 58 BRCA1 carriers (8.6%) were diagnosed with an occult carcinoma: two fallopian tube carcinomas, two ovarian carcinomas and one case was defined as a fallopian tube/ovarian carcinoma. No occult carcinomas were found in the other groups. Of the 38 patients, who underwent a bilateral oophorectomy (mean follow-up 45 months), three of 26 BRCA1 mutation carriers (3.4 in 100 women-years) developed peritoneal papillary serous carcinoma (PPSC) during follow-up. So far, no PPSC have occurred in the 90 women, who underwent a salpingo-oophorectomy (mean follow-up 12 months), including 58 BRCA1 carriers (0 in 60 in women-years). These results contribute to the thesis that BRCA1 germline mutation carriers are not only at risk for ovarian cancer, but also for fallopian tube carcinoma and peritoneal papillary serous carcinoma. Our data suggest that PPSC risk among BRCA2 carriers is lower than among BRCA1 carriers.

Women with a BRCA1 or BRCA2 germline mutation are at an increased risk of developing breast and ovarian cancer. At the age of 70, the cumulative risk of developing ovarian cancer in a BRCA1 mutation carrier ranges from 16 to 85%, whereas the risk with a BRCA2 mutation is 10–27% (Ford *et al*, 1994, 1998; Easton *et al*, 1995; Burke *et al*, 1997; Struewing *et al*, 1997). Annual screening by assessing serum CA-125 and transvaginal ultrasound monitoring is recommended in these patients (Burke *et al*, 1997). Since the efficiency of these screenings methods is still not clear (Laframboise *et al*, 2002), prophylactic surgery of the ovaries for patients with proven BRCA1 or BRCA2 mutations or a strong family history of breast and/or ovarian cancer is an important option (Eisen *et al*, 2000; Rebbeck *et al*, 2002). It has been found that this procedure reduces the risk of ovarian cancer by 96% and breast cancer by 53% in BRCA1 or BRCA2 mutation carriers (Kauff *et al*, 2002; Rebbeck *et al*, 2002).

Fallopian tube carcinoma comprises 0,.5% of all gynaecologic tumours (Schneider *et al*, 2000). The true incidence is probably underestimated, since most patients diagnosed with fallopian tube carcinoma present with more advanced disease involving the tubes as well as the ovaries and are classified as ovarian cancer. Primary fallopian tube carcinoma can only be diagnosed when the largest bulk of the carcinoma is present in the fallopian tube, or if a transition of dysplastic tubal epithelium to carcinoma is observed (Woolas *et al*, 1997). In cases where there is no evidence which favours either a fallopian tube carcinoma or an ovarian carcinoma, the term tubo-ovarian carcinoma is used (Scully *et al*, 2003). Fallopian tube cancer has been reported in BRCA1 and BRCA2 germline mutation carriers recently (Schubert *et al*, 1997; Tong *et al*, 1999; Hartley *et al*, 2000; Rose *et al*, 2000; Sobol *et al*, 2000; Zweemer *et al*, 2000; Agoff *et al*, 2002).

There is a growing number of manuscripts reporting the incident findings of tumours at prophylactic salpingo-oophorectomy, including patients with fallopian tube cancer (Salazar *et al*, 1996; Hartley *et al*, 2000; Lu *et al*, 2000; Colgan *et al*, 2001; Paley *et al*, 2001; Agoff *et al*, 2002; Kauff *et al*, 2002; Leeper *et al*, 2002; Rebbeck *et al*, 2002). However, these are either small studies including no more than 60 patients or studies without a clear distinction between either salpingo-oophorectomy *vs* an oophorectomy, or BRCA1 *vs* BRCA2 carriers. The development of peritoneal papillary serous carcinoma (PPSC) has also been observed after prophylactic surgery of the ovaries. (Tobacman *et al*, 1982; Chen *et al*, 1985; Lynch *et al*, 1986, 1991; Piver *et al*, 1993; Kauff *et al*, 2002; Rebbeck *et al*, 2002). No comparison has been made between the outcome of a bilateral oophorectomy and the outcome of a bilateral salpingo-oophorectomy.

The purpose of this study is to assess the prevalence of histopathologic findings at bilateral prophylactic (salpingo)- oophorectomy and incidence in follow-up based on the findings of 128 high-risk women.

## PATIENTS AND METHODS

### Patients

Clinical data of women who had undergone a prophylactic oophorectomy or salpingo-oophorectomy at the Antoni van Leeuwenhoek Hospital were obtained from clinical charts. The women were determined to be at high risk by the following criteria: BRCA1 or BRCA2 germline mutation carriers or women with breast cancer from a hereditary breast-ovarian cancer (HBOC) family. DNA testing was either done before the prophylactic surgery or performed after testing became available. If no mutation was found in a person herself or in her family, the DNA result was called non-informative. Women were seen by a gynaecologic oncologist once a year for a gynaecologic examination, transvaginal ultrasound and serum CA-125 determination, also after the prophylactic surgery for screening of papillary serous peritoneal cancer.

From January 1990 to November 2001, we identified 38 women (mean age 47 years) who had undergone prophylactic oophorectomy, and 90 patients (mean age 46 years) who received prophylactic salpingo-oophorectomy (Table 1

**Table 1:** Patient characteristics of 38 patients with a bilateral oophorectomy and 90 patients with a bilateral salpingo-oophorectomy

	**Bilateral oophorectomy**		**Bilateral salpingo-oophorectomy**	
**Characteristics**	***N*=38**	**%**	***N*=90**	**%**
Previous breast cancer	28	74	55	61
Laparoscopic surgery	24	63	59	66
Laparotomic surgery	14	37	31	34
BRCA1 mutation	26	68	58	64
BRCA2 mutation	3	8	6	7
BRCA1 and 2 mutation	0	0	1	1
Non-informative DNA test result	9	24	25	28

). In May 1997, an incidental salpingo-oophorectomy was performed, while the standard procedure up till that time was a bilateral oophorectomy. In this patient, an occult fallopian tube carcinoma was found. This finding, together with the growing number of articles about salpingo-oophorectomy, prompted us to extend the prophylactic procedure from oophorectomy to salpingo-oophorectomy. In all, 35 patients underwent a primary laparotomic bilateral oophorectomy, 93 a laparoscopy, from which 10 operations were converted into a laparotomy. The mean age of all women was 46 years, range 26–74 years.

### Pathology

After oophorectomy or salpingo-oophorectomy, the ovaries and fallopian tubes were entirely sectioned. Occult carcinoma was defined as follows: the patients were not suspected of having an ovarian malignancy before surgery, determined by patient history, pelvic examination, transvaginal examination or serum CA-125 determination, and the tumour was an unexpected finding seen only at histological examination. The histological diagnosis fallopian tube carcinoma was based on tumour size, histological markers and histological subtype. To study the relation between normal fallopian tube tissue and the carcinoma in a BRCA1 mutation carrier, a molecular analysis was performed in one patient. DNA was isolated by micro dissecting areas of 10 *μ*m and extracted in a solution of TNE buffer, 500 mM EDTA and 20 mg ml^−1^ proteinase K. The isolated DNA was analysed for BRCA1 loss of heterozygosity (LOH) by polymerase chain reaction using 10 microsatellite markers, six spanning chromosome 17 and four spanning other chromosomes. P53 staining was performed in all cases with a carcinoma, because BRCA1 germline mutation carriers with breast cancer were more likely to be positive for p53 protein compared with sporadic breast cancer patients (Lakhani *et al*, 2002). P53 expression was detected immunohistochemically using D0–7 monoclonal antibodies (DAKO, Denmark) and scored as overexpressed when staining was present in more than 50% of tumour cells.

## RESULTS

Characteristics of the 38 women (mean age 47 years, range 31–64) who underwent an oophorectomy and the 90 women (mean age 46 years, range 26–74) who underwent a salpingo-oophorectomy are depicted in Table 1. There was no difference between the two groups in terms of percentage with BRCA1 or BRCA2 mutations or percentage with a history of breast cancer.

In the group of women who underwent an oophorectomy, no occult carcinomas were found. In the second group of women with a salpingo-oophorectomy, five occult tumours were found in 8.6% of 58 BRCA1 mutation carriers (Table 2

**Table 2:** Clinical data of patients with occult carcinoma at salpingo-oophorectomy (patients 1–5) and PPSC (patients 6–8) after oophorectomy

**Patients diagnosed at prophylactic surgery**	**BRCA status**	**Age (year)**	**Diagnosis (stage)**	**Histology**	**P53 staining**	**Status**	**Follow-up (months)**
1	3875del4(BRCA1)	33	Fall tube (stage 1a)	Endometrioid adenoca gr II	Negative	NED	46
2	BRCA1*	49	Fall tube (stage 3b)	Serous papillary adenoca gr II	Positive	Rec.	20
3	2312del5(BRCA1)	45	Fall tube/ovary (stage 4)	Adenoca gr III	Positive	Rec.	11
4	1411insT(BRCA1)	47	Ovary (stage 1c)	Serous papillary adenoca gr II	Positive	NED	35
5	Exon11stop(BRCA1)	37	Ovary (stage 1a)	Serous papillary adenoca gr II	Positive	NED	11
							
**Patients diagnosed in follow-up**	**BRCA status**	**Age (year)**	**Diagnosis (stage)**	**Histology**	**P53 staining**	**Status**	**Follow-up (months)**
6	Exon22del(BRCA1)	60	PPSC (stage 3c)	Serous papillary adenoca gr I	Positive	Rec.	22
7	BRCA1*	53	PPSC (stage 3c)	Adenoca gr III	Negative	DOD	53
8	BRCA1*	64	PPSC (stage 3c)	Serous papillary adenoca gr III	Positive	NED	20

gr=grade; Fall=Fallopian; adenoca=adenocarcinoma; NED=no evidence of disease; Rec=recurrence; PPSC=peritoneal papillary serous carcinoma; DOD=dead of disease;

* Not further specified.

): two cases of fallopian tube carcinoma, one case of fallopian tube/ovarian carcinoma and two cases of ovarian carcinoma. The patients with an occult tumour had a mean age of 42.2 years (range 33–49). All five tumours were only detected at microscopic pathological examination. At subsequent staging procedures, there were two FIGO stage 1A, one stage 1C, one 3B and one stage 4 carcinomas. Despite adjuvant chemotherapy, two patients (patient 2 and 3) developed a recurrence after 11 and 20 months.

**Figure 1:**
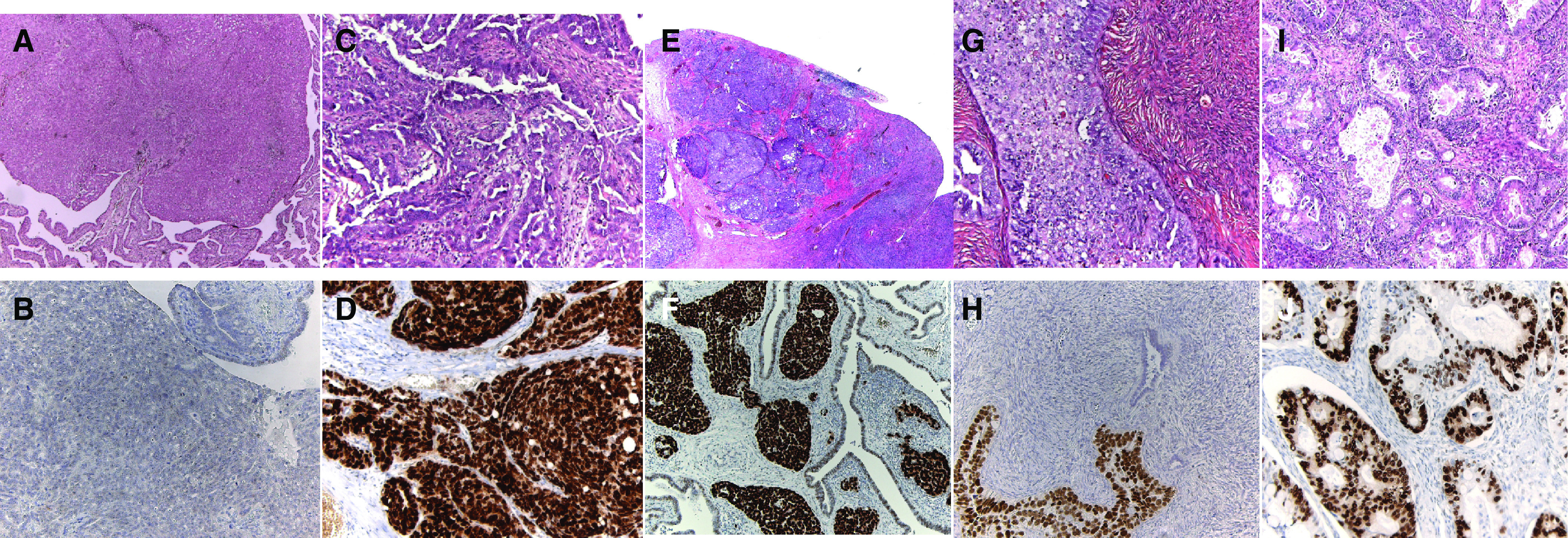
Endometrioid adenocarcinoma (case patient 1), obliterating the lumen of the fallopian tube with a diameter of 2.5 mm (haematoxylin (H&E)), magnification × 50). (**B**) Negative P53 staining of the same tumour in (**A**) (magnification × 200). (**C**) Microscopic serous papillary adenocarcinoma (case patient 2), 1.0 cm in diameter (H&E, magnification × 100). (**D**) Overexpression of P53 of the same tumour in (**C**) (magnification × 200). (**E**) Poorly differentiated adenocarcinoma (case patient 3), 5 mm in diameter (H&E, magnification × 25). (**F**) Positive P53 staining of the same tumour in (**E**) (magnification × 200). (**G**) Invasive serous papillary adenocarcinoma (case patient 4), with a diameter of 8 mm. Note the formation of complex papillae (H&E, magnification × 100). (**H**) The same tumour as (**H**), P53 positive (magnification × 200). (**I**) Serous papillary adenocarcinoma (case patient 5) with mitotic activity (H&E, magnification × 100). (**J**) The same tumour as (**I**), P53 positive (magnification × 200).

The microscopical examination of patient 1 showed a tumour embolus with a diameter of 2.5 mm nearly completely obliterating the lumen of the left fallopian tube (Figure 1

). P53 staining was negative. To examine the role of BRCA1 in fallopian tube carcinoma development in this patient, DNA was isolated from the tumour and from the surrounding normal tissue for a BRCA1 LOH analysis. Six markers were selected located in the vicinity of the BRCA1 locus at chromosome 17. An LOH of the nonmutated BRCA1 allele was detected in the fallopian tube tissue. The patient is alive and well after 46 months of follow-up.

At 1 year after treatment for breast cancer with surgery, adjuvant chemotherapy and tamoxifen, patient 2 underwent prophylactic salpingo-oophorectomy. Only at microscopy, a primary serous papillary adenocarcinoma (diameter 10 mm) at the end of the fallopian tube was detected. Overexpression of p53 was observed. Despite postoperative chemotherapy, a recurrence was noted after 20 months. The pathology of patient 3 revealed a poorly differentiated adenocarcinoma spread diffusely in both fallopian tubes and one ovary (diameter 5 mm). In the lymphatic vessels of the fallopian tubes tumour fragments were noticed. However, the bulk of the tumour was seen on the surface of the ovary as well as both tubes. The diagnosis ‘fallopian tube/ovarian carcinoma’ was made. The tumour cells were p53 positive. At 11 months after confirming the initial diagnosis, she had a recurrence. Patient 4 was treated 3 years before for breast cancer. At prophylactic surgery, an occult ovarian carcinoma with a diameter of 8 mm was seen at microscopy. The histology of patients 4 and 5 revealed a grade two papillary serous ovarian adenocarcinoma and p53 staining was positive in both cases. The tubes were all normal. They received no further treatment and are alive without recurrence.

The mean follow-up of the 38 women who underwent bilateral oophorectomy was 45 months (range 24.1–93.1 months). Three cases of papillary serous peritoneal cancer were diagnosed 27, 33 and 70 months after prophylactic oophorectomy in 26 BRCA1 carriers (3.4 in 100 BRCA1 women-years). At prophylactic surgery, a right-sided salpingo-oophorectomy was performed in patient 6, since the left tube could not be recognised due to adhesions as a result of culdotomic sterilisation. Due to a rising CA-125 27 months later, she underwent an exploratory laparatomy and was found to have peritoneal papillary serous carcinoma. The tumour cells were p53 positive. The left fallopian tube could not be identified. The debulking was suboptimal. Despite a complete remission after six paclitaxel/cisplatin cycles, she had a recurrence 22 months later. In the other two patients, only the ovaries had been removed at prophylactic surgery. The tubes were left *in situ* and the pathology reports showed no evidence of ovarian cancer. Patient 7 had also a rising CA-125 33 months after the prophylactic surgery. She had a debulking. The pathologist saw no fragments of tumour intraluminal in the remnant tubes. P53 staining was negative. Despite a complete remission after six paclitaxel/cisplatin cycles, she died of liver metastases 53 months after the diagnosis. Patient 8 had a distended abdomen due to ascites 70 months after the oophorectomy. Serum CA-125 was also elevated. She also had a debulking, where only the left tube could be identified. The tube showed an intact architecture microscopically. The serous papillary tumour cells were p53 positive. There is still no evidence of disease 20 months after completion of six cycles of paclitaxel/carboplatin.

No cases of papillary serous peritoneal cancer were registered in the follow-up after the group with a bilateral salpingo-oophorectomy including 58 BRCA carriers (0 in 60 women-years). However, the mean follow-up of all these women was still short (12 months, range 0.5–65.5).

## DISCUSSION

In our series, five occult tumours were found in 58 BRCA1 germline mutation carriers (8.6%), who had undergone prophylactic salpingo-oophorectomy (Table 2). None of the tumours were suspected before or at the time of surgery; all five carcinomas were only seen at microscopy. No occult tumours were found in the remaining women (one BRCA1 and BRCA2, six BRCA2 and 25 women with non-informative test results, respectively) who underwent a bilateral salpingo-oophorectomy, nor in the group of 38 women (26 BRCA1, three BRCA2, nine women with non-informative test results), who received a bilateral oophorectomy. PPSC during follow-up was found in the BRCA1 carriers who underwent an oophorectomy (3.4 per 100 women-years), and none in the other groups.

Occult carcinomas have been reported before. Colgan *et al* found five occult carcinomas of the ovaries and/or *in situ* or invasive carcinoma of the fallopian tube among 60 patients (mean age 48.5 years), all BRCA1 mutation positive. The prevalence of occult tumours found in their series at prophylactic surgery in 27 BRCA1 mutation carriers is 18.5%. In the study of Lu *et al*, four (12%) of 33 women (mean age 46 years) with a high calculated risk of carrying a BRCA1 or BRCA2 mutation, who had undergone prophylactic salpingo-oophorectomy, had occult ovarian cancer found only at pathological examination (Lu *et al*, 2000). Three women had a BRCA1 mutation, while one patient carried a BRCA2 mutation. Recently, Kauff *et al* described three cases (3.0%) of unexpected findings at total abdominal hysterectomy/bilateral salpingo-oophorectomy in 101 BRCA1 or BRCA2 germline mutation carriers (mean age 47.5 years). One fallopian tube carcinoma and two ovarian carcinomas were diagnosed. A prevalence of 2.3% occult ovarian tumours was found by Rebbeck *et al* in their series of 259 mutation carriers (mean age 42.0 years) undergoing oophorectomy or salpingo-oophorectomy. Thus, a prevalence of 2.3–18.5% of occult tumours in BRCA1 or BRCA2 germline mutation carriers has been found. First, this wide range is probably attributable to the variable sizes of cohorts. Second, our finding of 8.6% prevalence of occult tumours is established in BRCA1 mutation carriers undergoing prophylactic salpingo-oophorectomy, while other series did not make a clear distinction between a salpingo-oophorectomy or an oophorectomy, nor between BRCA1 and BRCA2 carriers. Third, the risk of developing ovarian or fallopian tube carcinoma increases with age; so the low prevalence found by Rebbeck *et al* might be due to a lower mean age at prophylactic surgery. These studies show the importance of attentiveness of occult tumours. It is interesting to see that no occult carcinomas were found in our remaining group of women with either a BRCA2 mutation or non-informative DNA test results. Our results suggest that carriers of a BRCA1 germline mutation have a substantial higher risk of occult carcinomas compared to BRCA2 carriers or non-informative test results. However, the power for the group of BRCA2 mutation carriers is still low.

To estimate the prevalence of occult fallopian tube and ovarian carcinoma at prophylactic salpingo-oophorectomy, we need to be able to distinguish both carcinomas, which is often not possible in advanced disease. In our series, two clear cases of fallopian tube carcinoma and one fallopian tube/ovarian carcinoma were found. In patient 3, the exact origin was not clear: the bulk of the tumour was seen on the surface of the ovaries and both tubes. The histological subtype ‘poorly differentiated adenocarcinoma’ was also not helpful, since this cell type is associated with both tumours (Scully *et al*, 2003).

Zweemer *et al* (2000) showed a loss of the wild-type BRCA1 allele in two fallopian tube carcinomas. In both patients, the presence of a BRCA1 mutation was confirmed and a loss of the wild-type BRCA1 allele in both tumours was shown. In our first patient, we used the same method and also found a LOH of the nonmutated BRCA1 allele in the fallopian tube tissue. These findings strongly suggest that fallopian tube cancer is linked to BRCA1 mutations.

Paley *et al* (2001) described two patients with occult fallopian tube carcinomas at surgical prophylaxis, one carcinoma *in situ* without extension to the stroma and the other patient with a papillary serous adenocarcinoma without extension to the serosa. It was advised that hysterectomy should be discussed with patients who are considering prophylactic salpingo-oophorectomy. However, there are no long-term data to support hysterectomy in addition to bilateral salpingo-oophorectomy. Until now, just a few reports were published linking BRCA1 and 2 mutations with uterine serous papillary carcinomas (Hornreich *et al*, 1999; Lavie *et al*, 2000). Furthermore, hysterectomy has additional risks and complications (Dicker *et al*, 1982). Given the anatomy, a hysterectomy cannot prevent the development of PPSC. Piver *et al* (1993) described nine cases of PPSC after abdominal total hysterectomy and salpingo-oophorectomy.

All five occult carcinomas were discovered only at microscopical examination. Neither transvaginal examination nor serum CA-125 determination was sufficient enough to detect these malignancies. The combination of CA-125 and TVU was tested before in the general population (Jacobs *et al*, 1999; Menon *et al*, 1999) and was shown to be a feasible screenings method. However, failure to detect more than 57% of early-stage disease is cited as the major limitation of this type of screening (Jacobs *et al*, 1993). More studies are required to establish in a large group of high-risk patients the sensitivity, specificity and predictive value of ovarian cancer screening, by means of pelvis examination, transvaginal ultrasound and serum CA-125 determination. This high failure rate of ovarian screening stresses the importance of complete enclosure of both ovaries and tubes following prophylactic surgery in order not to miss the presence of an occult carcinoma. We agree with Colgan *et al* (2001) who advised to entirely section both tubes and ovaries.

Four of the five occult tumours and two papillary serous peritoneal carcinomas of women with a BRCA1 germline mutation were positive of P53 protein. These findings are consistent with the results of Lakhani *et al*, who found that breast carcinomas in patients with a BRCA1 mutation are more likely to be positive for p53 protein than controls.

Among the 38 patients who underwent a bilateral oophorectomy were 26 BRCA1 mutation carriers, three of whom (3.4 in 100 women-years) developed papillary serous carcinoma of the peritoneum 27, 33 and 70 months after the prophylactic surgery. The lack of PPSC incidence in the salpingo-oophorectomy group including 58 BRCA1 carriers (0 in 60 in women-years) could well be explained by the difference in follow-up duration (45 months *vs* 12 months in the salpingo-oophorectomy group, respectively). Only 10 women of the group with a salpingo-oophorectomy passed the time point at which the first PPSC case occurred in the group of women with a bilateral oophorectomy. For a meaningful comparison between the two types of surgery, a longer follow-up is needed. Another explanation of the difference of PPSC incidence may be that PPSC could be a metastasis of the remnant fallopian tubes. However, at the time of the diagnosis of PPSC no malignant lesions were found in the fallopian tubes. Furthermore, it has been shown that PPSC has developed after salpingo-oophorectomy (Piver *et al*, 1993). So, we have concluded that PPSC is a primary tumour and not a metastasis. Kauff *et al* reported one case of PPSC (0.5 in 100 women-years) in 98 BRCA1 and BRCA2 mutation carriers, who chose risk-reducing salpingo-oophorectomy with a mean follow-up duration of 23.4 months. The incidence of PPSC in our study (3.4 in 100 BRCA1 women-years) was higher than the 0.5 in 100 women-years, but this may be due to a longer mean follow-up duration (45 months in our study *vs* 23.4 months in the study by Kauff *et al*). Second, the later study also includes 42 BRCA2 carriers (43%), while the incidence in our series is established for BRCA1 carriers who have a higher intrinsic ovarian cancer risk, and this may also be true for PPSC. Other studies neither described procedures nor whether the cases were BRCA1 or BRCA2 mutation carriers (Piver *et al*, 1993; Rebbeck *et al*, 2002). Our series of 64 BRCA1 and BRCA2 mutation carriers after salpingo-oophorectomy includes only six BRCA2 carriers (9.4%). The only report of PPSC occurring in two BRCA2 carriers after hysterectomy and salpingo-oophorectomy is published recently (Foulkes *et al*, 2003). Unfortunately, no data on follow-up of a cohort are given. Six BRCA2 mutation carriers are found in 22 cases of PPSC by Levine *et al* (2003), but whether these cases developed after a prophylactic procedure is not mentioned. Clearly, it is too early to conclude that BRCA2 carriers face a lower risk than BRCA1 carriers of developing PPSC.

These results contribute to the thesis that BRCA1 germline mutation carriers are not only at risk for ovarian cancer but also for fallopian tube carcinoma and peritoneal papillary serous carcinoma. Prophylactic salpingo-oophorectomy and sectioning both tubes and ovaries is recommended in order to not miss any occult carcinomas. Our data suggest that PPSC risk among BRCA2 carriers is lower than among BRCA1 carriers.
